# Temperature-Resolved Crystallography Reveals Rigid-Body Dominance over Local Flexibility in B‑Factors

**DOI:** 10.1021/acsomega.5c04454

**Published:** 2025-08-20

**Authors:** Fernando de Sá Ribeiro, Luís Maurício T. R. Lima

**Affiliations:** † Laboratório de Biotecnologia Farmacêutica (pbiotech), Faculdade de Farmácia, 28125 Universidade Federal do Rio de Janeiro, Rio de Janeiro, RJ 21941-902, Brazil; ‡ Programa de Pós-Graduação em Química Biológica, 28125 Universidade Federal do Rio de Janeiro, Rio de Janeiro, RJ 21941-902, Brazil; § Programa de Pós-Graduação em Ciências Farmacêuticas, Faculdade de Farmácia, 28125 Universidade Federal do Rio de Janeiro, Rio de Janeiro, RJ 21941-902, Brazil; ∥ Programa de Pós-Graduação em Nutrição, 28125 Universidade Federal do Rio de Janeiro, Rio de Janeiro, RJ 21941-902, Brazil

## Abstract

The crystallographic B-factor (Bf), also known as the Debye–Waller factor (DWF) or temperature factor, relates to the mean-square displacement of the atoms (X^2^). X^2^ may be composed of individual contributions from lattice disorder (LT), static conformational heterogeneity (H) throughout the lattice, rigid body vibration (RB), local conformational vibration (V), and zero-point atomic fluctuation (A). The Bf has been widely employed as a surrogate measure of local protein flexibility, although such relation has not been confirmed. In addition, reproducibility of the absolute B-factor is difficult to achieve, hampering the understanding of their individual contribution. Here, we report the crystallographic investigation of the enzyme–ligand complex of trypsin with benzamidine from cryo to room temperature, through a 200 K range (9-point triplicate design), by crystal stabilization with hydrophobic grease. The extent of temperature-induced conformational changes showed no connection with their respective B-factors. The B-factor variation due to temperature was constant for all atoms of the system, of about 0.005 K^–1^. The results caution against interpreting absolute, normalized, or zero-point B-factors as direct proxies for protein dynamics, which is further supported by structural analysis of data from independent groups with trypsin–benzamidine complexes obtained under dissimilar experimental conditions. The similar thermal dependence of the B-factor for all atoms of the system suggests a major contribution of this physical variable over uniform rigid body vibration.

## Graphical abstract

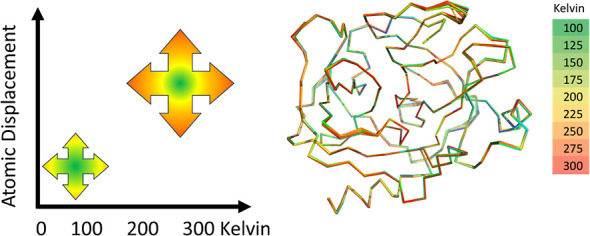

## Introduction

The molecular properties of proteins have been studied for over 60 years as celebrated by the seminal work of Perutz^1^ and Kendrew^2^ in the elucidation of the crystal structure of hemoglobin and myoglobin, respectively. Conformational transitions of proteins upon physical and chemical variables have been investigated for close to a century since the pioneer work of William Astbury and Dorothy (Crowfoot) Hodgkin^3,4^ and the demonstration by X-ray diffraction of protein conformational transition between α-rich and β-rich structures.

Molecular dynamics lies between molecular snapshots and conformational transitions and are essential for deeper understanding of mechanisms.^5^ Dynamics is an essential part of life at all scales, from atoms to molecules, organisms, and the biome. Motion can also occur on different extensions and time scales. Atomic motion can take picoseconds within a few square Angstroms,^6^ while protein motion either functionally important (FIM) or biologically unimportant (BUM)^7^ can take nano to microseconds over tens of nanometers.

Molecular structural biology techniques such as single-crystal and serial crystallography, Nuclear Magnetic Resonance (NMR), cryoelectron microscopy (cryoEM), atomic force microscopy, molecular dynamics simulations along with spectroscopic technics including circular dichroism and Fourier-transformed infrared have been used in the understanding of the dynamics and conformational transitions.^8^ Among these techniques, crystal (single or multi) diffraction (X-ray, electron) is yet routinely used in the elucidation of polypeptide structures and is highly reproducible as shown by the protein obtained from diverse biotechnological processes and solved using data from different instruments (home sources and synchrotron).^9,10^ Along with coordinates, the crystallographic structures also provide a less reproductible parameter, the B-factor, which is known as the temperature factor or the atomic displacement parameter,^11–14^ and reflect the mean-square atomic displacement (*X* ^2^) by the Debye–Waller function (DWF) as follows:

1 $$B=8\pi^{2}X^{2}$$

where *X* ^2^ can arise from various sources, with contribution from zero-point (zero Kelvin) atomic fluctuation (*X* ^2^_A_), conformational vibration (*X* ^2^_V_), static conformational heterogeneity (*X* ^2^_H_), rigid body vibration (*X* ^2^_RB_), and crystal lattice disorders (*X* ^2^_LT_).^15,16^

The lack of correlation between the B-factor and conformational changes induced by temperature challenges the popular use of the B-factor as a surrogate for protein dynamics. In fact, the dissociation between B-factor and dynamics has been anticipated close to 30 years ago,^17,18^ but is still under debate, and limited by the reproducibility of B-factor measurements.

The large variability of B-factor between measurements, crystals, and setups can be empirically scaled down by its average B-factor (*B*_avg_), resulting in a reproducible normalized B-factor (*B*_norm_)^11,19^ in the form of

2 $$B_{norm}=B_{obs}/B_{avg}$$

While mathematical normalization provides comparability between data sets, it is desirable to measure absolute (raw) B-factor once ensured that the crystal is stabilized, allowing the investigation of temperature as a continuous variable from cryogenic to high (above room) temperature^20^ overcoming the critical temperature range of about ∼210 K which relates proteins to glasses.^7^

In order to obtain further insight into the linkage between the B-factor and conformational transition, we used trypsin in complex with benzamidine as a case study. Trypsin–benzamidine crystal structure has been shown to be highly reproducible at high resolution.^10^ In this study, we investigated the temperature dependence of B-factor over temperature from 100 to 300 K in triplicate at each temperature, using trypsin–benzamidine crystals protected with hydrocarbon grease mounted in regular nylon loops, which has the advantage of not using sophisticated systems such as capillaries and tubbing.^21^ We discuss our findings in light of potential correlation between observed conformational changes and B-factors.

## Materials and Methods

### Materials

Bovine pancreatic trypsin (Cat no. SLBZ8570) and benzamidine (Cat no. MKCH1700) were obtained from Merck/Sigma-Aldrich and kept at −20 °C until use. All other reagents were of analytical grade.

### Methods

#### Protein Crystallography

Trypsin crystals were prepared via vapor diffusion using the sitting-drop method on Corning 3552 plates. Each drop consisted of 1.0 μL of 35 mg/mL Trypsin freshly prepared by dissolving the protein with 5 mg/mL Benzamidine, 100 mM Hepes, and 3 mM CaCl_2_ at pH 7.0, combined with 1 μL of a precipitating agent. The drops were equilibrated against 80 μL of a reservoir solution made up of 0.2 M K_2_HPO_4_ and 20% w/v Polyethylene glycol 3,350 at a temperature of 22 ± 2 °C. Crystals suitable for diffraction emerged within 24 h and were harvested after 48 h. The crystals were manipulated using 20 μm nylon CryoLoops (Hampton Research). Each crystal was immersed in Apiezon N hydrocarbon grease and subsequently mounted on the goniometer in a nitrogen stream at the target temperature for data collection.

#### Diffractometer Setup and Data Acquisition

The crystals underwent X-ray diffraction and data collection by using CuKα radiation with a constant exposure time. This was performed using a 30 W air-cooled μS microfocus source (Incoatec) attached to a D8-Venture diffractometer (Bruker) at the CENABIO-UFRJ facility, operating at 50 kV and 1.1 mA. The data were captured on a Photon II detector (Bruker). Crystals were maintained under a nitrogen stream at the specified temperature with a flow rate of 1.2 L/h, regulated by a CryoStream 800 instrument (Oxford Cryogenics). All data sets were obtained with 30 s exposures and 0.5° oscillation per image, ensuring at least 99% completeness by assuming identical Friedel pairs and aiming for a resolution of 1.5 Å.

#### Data Processing and Analysis

The data were collected, indexed, integrated, and scaled using Proteum3 (Bruker AXS Inc.), followed by analysis with Truncate (C.C.P.4 v7.0.071). The crystal structures were solved through molecular replacement, involving 20 cycles of rigid body search with RefMac v5.8.0238, employing PDB entry 1S0R (Bovine Pancreatic Trypsin inhibited with Benzamidine at Atomic resolution, at 1.02 Å). This process yielded a definitive solution for a monomer in the asymmetric unit. The initial solution underwent further refinement with 10 cycles of restrained refinement by using Refmac. Real space refinement was performed by visually inspecting both the map and the model with the C.O.O.T. v0.8.9.2, adjusting misplaced side chains, and adding water molecules at a 1.2 σ threshold. This was followed by an additional 10 cycles of restrained refinement using Refmac. The data processing workflow was conducted using default modes in order to avoid bias. Subsequent data analysis was conducted using Superpose version 1.05 from C.C.P.4. Global pairwise alignment of the trypsin–benzamidine structures was performed with ProSMART, and the analysis of the crystallographic B-factor was conducted using Baverage (CCP4). Refinements were also performed in default mode using Phenix-refine (Supporting Information).

The crystallographic information on data collection and refinement statistics is given in the Supporting Information (Table S1). Figures of crystal structures were generated using PyMOL v2.0. The atomic coordinates have been deposited in the Protein Data Bank (https://www.rcsb.org/), and the corresponding codes are provided in the Supporting Information. Reproducibility in use of different X-ray diffractometer setups and data processing workflows was previously validated,^19^ and thus we focused in a single workflow in this study.

#### RCSB Data Analysis

A curated set of information about the orthorhombic (*P*2_1_2_1_2_1_) trypsin crystals with benzamidine, determined via single-crystal X-ray diffraction, was retrieved from The Research Collaboratory for Structural Bioinformatics Protein Data Bank (RCSB PDB, rcsb.org) as of May 2024 (Supporting Information). After excluding entries with incomplete data, the remaining information was plotted for the intended analysis (Figure S5).

### Circular Dichroism

Thermal denaturation of trypsin–benzamidine (5 mg/mL) was conducted in a buffer containing 0.01 mg/mL Benzamidine, 10 mM Hepes, and 300 μM CaCl_2_ at pH 7.0, utilizing a circular dichroism spectropolarimeter (JASCO J-1000; Tokyo, Japan) equipped with a Peltier temperature control system from Jasco. Measurements were taken using a 100 μm path length quartz cuvette (Uvonic Instruments, Plainview, NY). Spectra were recorded over three scans from 260 to 190 nm, with a spectral bandwidth of 0.2 nm, a scan speed of 100 nm/min, and a response time of 1 s. Corresponding experiments with blank solutions were also performed to ensure accurate background subtraction. The temperature was increased at a rate of 5 °C/min, with a 60 s equilibration period before each measurement.

### Structural Analysis from RCSB-PDB

The RCSB was searched for crystallographic structures of bovine trypsin in complex with benzamidine in the space group *P*2_1_2_1_2_1_, resulting in 39 structures. The extracted values from these data sets included the average B-factor, Wilson B-factor, Rwork, Rfree, and cell parameters. Additionally, B-factors for Cα atoms and side chains were extracted and normalized according to previous studies. Finally, using a reference structure (PDB ID 9AW0), the structures were superimposed using Superpose v1.05 from C.C.P.4 (CCP4), and the RMSD for Cα and side chains was obtained.

### Data Analysis

Graphics were generated with GraphPad Prism v. 8.0.2 for Windows (GraphPad Software, San Diego, California USA, www.graphpad.com). Correlation analysis was performed using Pearson r and two-tailed distribution.

## Results

Reproducible crystal diffraction was performed at a high resolution from 100 to 300 K.

The crystallographic structure of trypsin bound to benzamidine was solved at high resolution (1.5 Å) over the temperature range of 100–300 K in 25 K intervals, with three data sets per temperature, from a total of 27 independent crystals. The use of hydrocarbon grease for crystal protection contributed to protection against solvent loss, resulting in reproducible structures and B-factors among different crystals collected at the same temperature and allowing collection over a wide range of temperature as a continuous variable. The crystal cell parameters showed only minor variations as a function of temperature (Figures S1 and S2). Circular dichroism (CD) measurements of the trypsin–benzamidine complex in 20 mM dibasic potassium phosphate buffer pH 7.0 confirmed the high thermal stability of the complex in solution, maintaining its structural integrity even at high temperatures (Figure S3).

### Temperature-Induced Conformational Changes

The alignment of trypsin structures demonstrated a high similarity between complexes at varying temperatures, as shown for backbone (Figure 1A) and side chains (Figure 1B), although with a progressive increase in global RMSD for both side chains (Figure 1C) and Cα (Figure 1D) and a lack of variability in average secondary structure content (Figure 1E).

**Figure 1:**
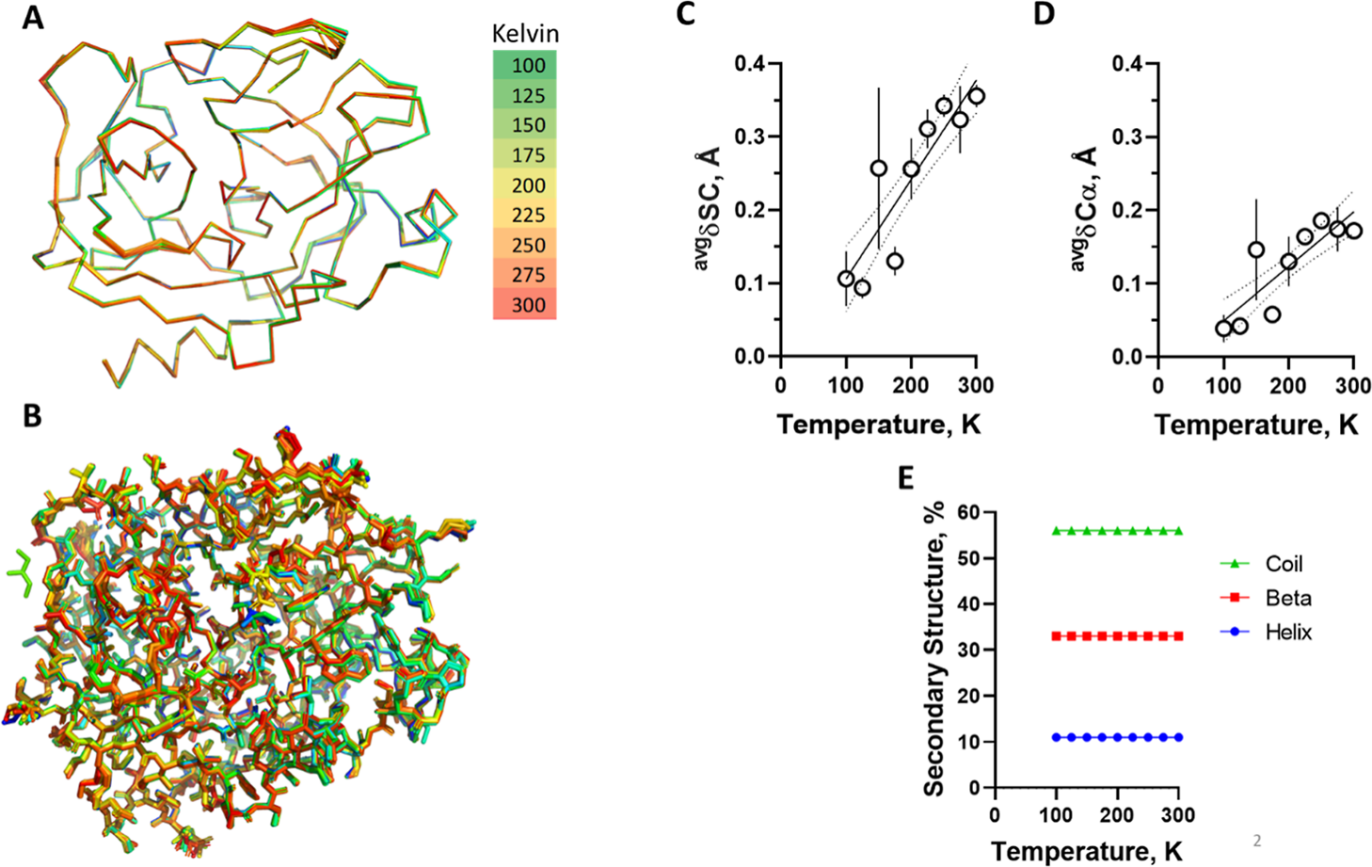
Global temperature-dependent conformational changes. Trypsin structure models solved from data collected at varying temperatures from 100 to 300 K were aligned using the reference structure (PDB: 9AVX) and are shown for (A) backbone and (B) side chains. Colors represent data collection temperature as indicated. *n* = 3 per temperature. Global (average values for residues 1–223) changes in conformation were inferred from superposition with a reference structure at 100 K. Continuous line is the first-order linear regression and dotted lines are 95% confidence interval. We observed a small (<1 Å) but progressive and significant increase in global conformational changes as inferred for distance changes in (C) SC (0.001358 Å.K^–1^; *p* ≤ 0.0001) and (D) Cα (0.0007422 Å.K^–1^; *p* ≤ 0.0001). The temperature changes have no variation in the percent of the secondary structure, as shown in (E). The symbol is the average, and the bar is the standard deviation (*n* = 3).

From the crystal structures, local conformational changes induced by the temperature were analyzed for both side chains (Figure 2A) and Cα (Figure 3A), with the latter showing a smaller amplitude of displacement. However, both chains exhibited nonuniform responsiveness to temperature, suggesting that temperature influences chain displacement in a localized manner.

**Figure 2:**
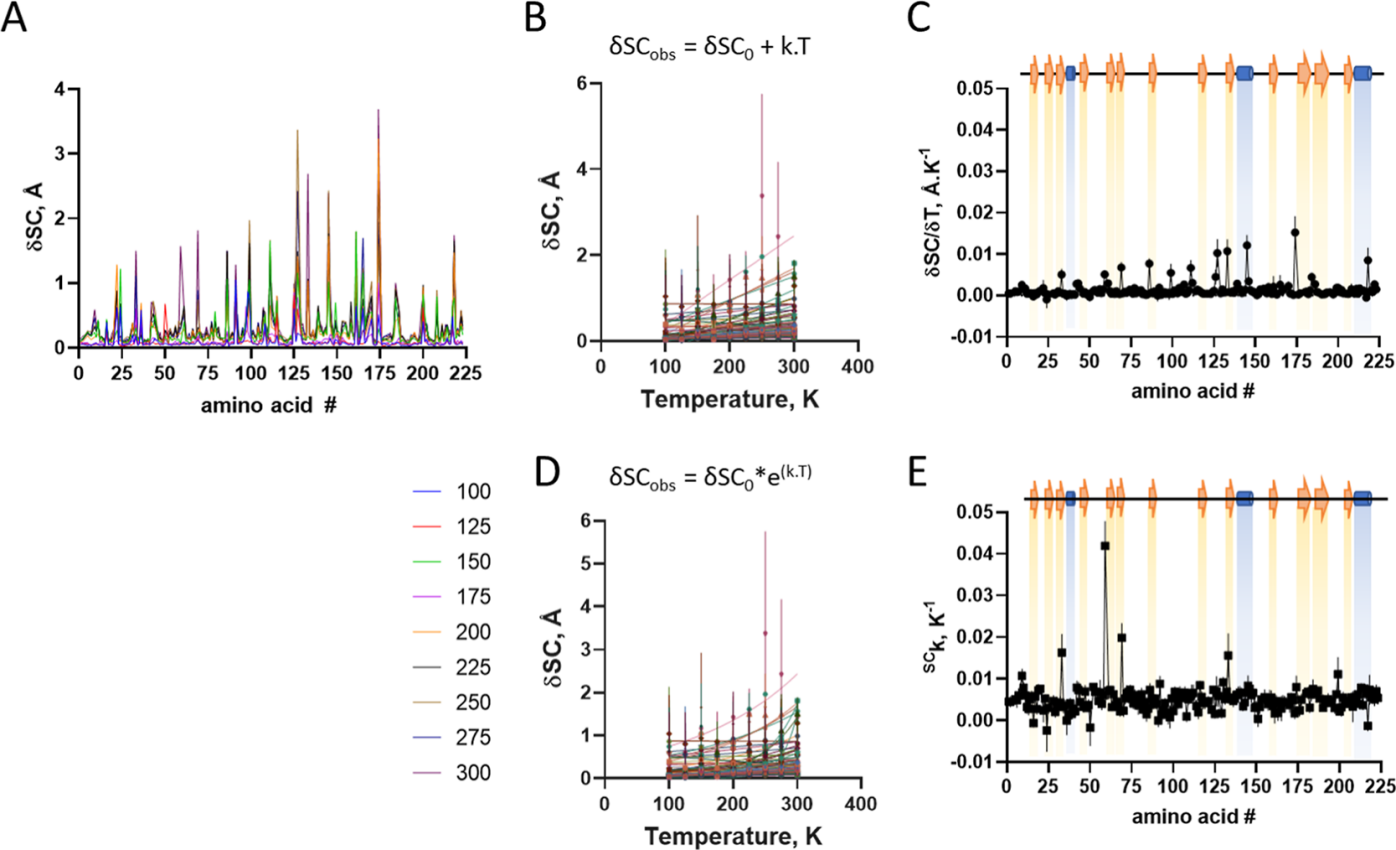
Temperature induced conformational changes in trypsin side chain. Structures were aligned with a reference structure at 100 K using Superpose (CCP4). (A) Changes in SC distances along protein sequence at varying temperatures (different colors). (B) Changes in SC distances as a function of temperature for each amino acid residue (different colors) protein sequence at varying temperatures. Lines are first order linear regression, from which were obtained their thermal-dependence of conformational change (δSC; angular coefficient of linear regression). (C) Distribution of thermal-dependent conformational change (δSC) along protein sequence. (D) Changes in Cα distances as a function of temperature for each amino acid residue (different colors) protein sequence at varying temperatures. Lines are exponential nonlinear regression, from which were obtained their exponential thermal constant (^δSC^k). (E) Distribution of exponential thermal constant (^δSC^k) of conformational change along protein sequence. Symbol is average and bar is standard deviation (*n* = 3).

**Figure 3:**
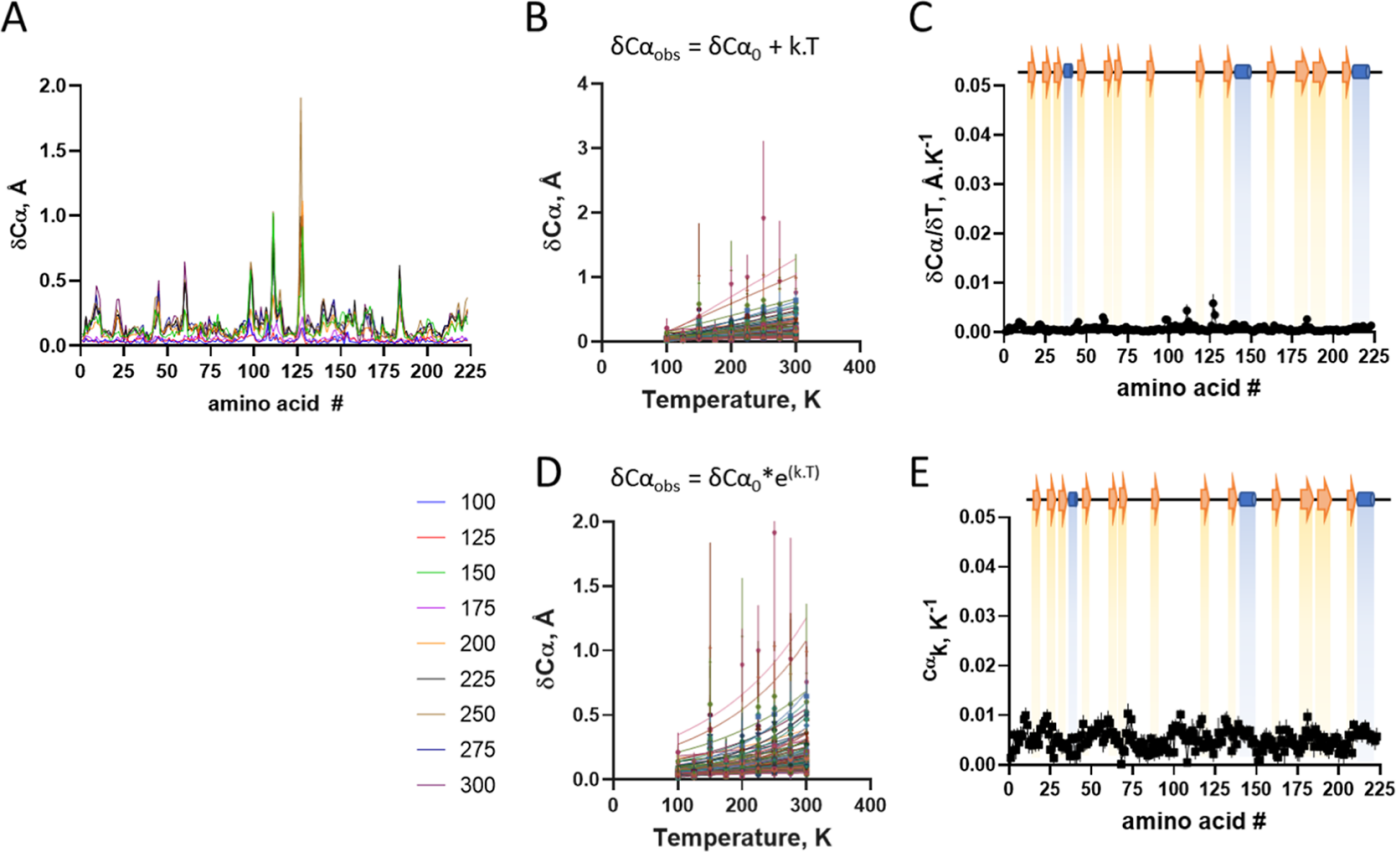
Temperature induced conformational changes in trypsin Cα. Structures were aligned with a reference structure at 100 K using Superpose (CCP4). (A) Changes in Cα distances along protein sequence at varying temperatures (different colors). (B) Changes in Cα distances as a function of temperature for each amino acid residue (different colors) protein sequence at varying temperatures. Lines are first order linear regression, from which were obtained their thermal-dependence of conformational change (δCα; angular coefficient of linear regression). (C) Distribution of thermal-dependent conformational change (δCα) along protein sequence. (D) Changes in Cα distances as a function of temperature for each amino acid residue (different colors) protein sequence at varying temperatures. Lines are exponential nonlinear regression, from which were obtained their exponential thermal constant (δCαk). (E) Distribution of exponential thermal constant (δCαk) of conformational change along protein sequence. Symbol is average and bar is standard deviation (*n* = 3).

The temperature-induced changes were analyzed by model-free regression, using first-order linear regression [eq 3] and nonlinear regression with a single exponential [eq 4] for Cα (Figure 2B–D) and side chains (Figure 3B–D)

3 $$\delta {SC}_{obs}=\delta {SC}_{0}+k.T$$

4 $$\delta {SC}_{obs}=\delta {{SC}_{0}}^{*}e^{\left(k.T\right)}$$

The temperature-dependence variation constants were plotted as a function of the polypeptide chain (Figures 2C–E and 3C–E and S4), and are shown to vary only marginally through the polypeptide chain (Figures 2C and 3C, respectively), suggesting the lack of major local propensity in conformational changes in response to temperature.

#### Global Changes in B-Factor by Temperature

We analyzed the average changes in B-factor as a function of temperature (Figure S4; Supporting Information). A positive exponential profile with temperature was observed for the B-factor derived from the Wilson plot (Figure 4A, and the average B-factor from the side chains (Figure 4B) and from the Cα (Figure 4C). The thermal dependence of these observed average B-factors was adjusted using the single exponential function [eq 5]­

5 $$B_{obs}=B_{0}\times e^{\left(k\times T\right)}$$

where *B*_**obs**_ is the refined B-factor, *B*_0_ is the B-factor extrapolated to zero Kelvin from regression, *k* is the thermal constant, and *T* is the data collection temperature (in Kelvin). This adjustment resulted in close average thermal factors of about ^avg^ *k* = 0.003944 K^–1^ and in ^avg^ *B*_0_ of about 5.63 Å^2^ for the B-factor dependence on temperature in the model-independent data from the Wilson plot and from the protein models (side chains and Cα).

**Figure 4:**
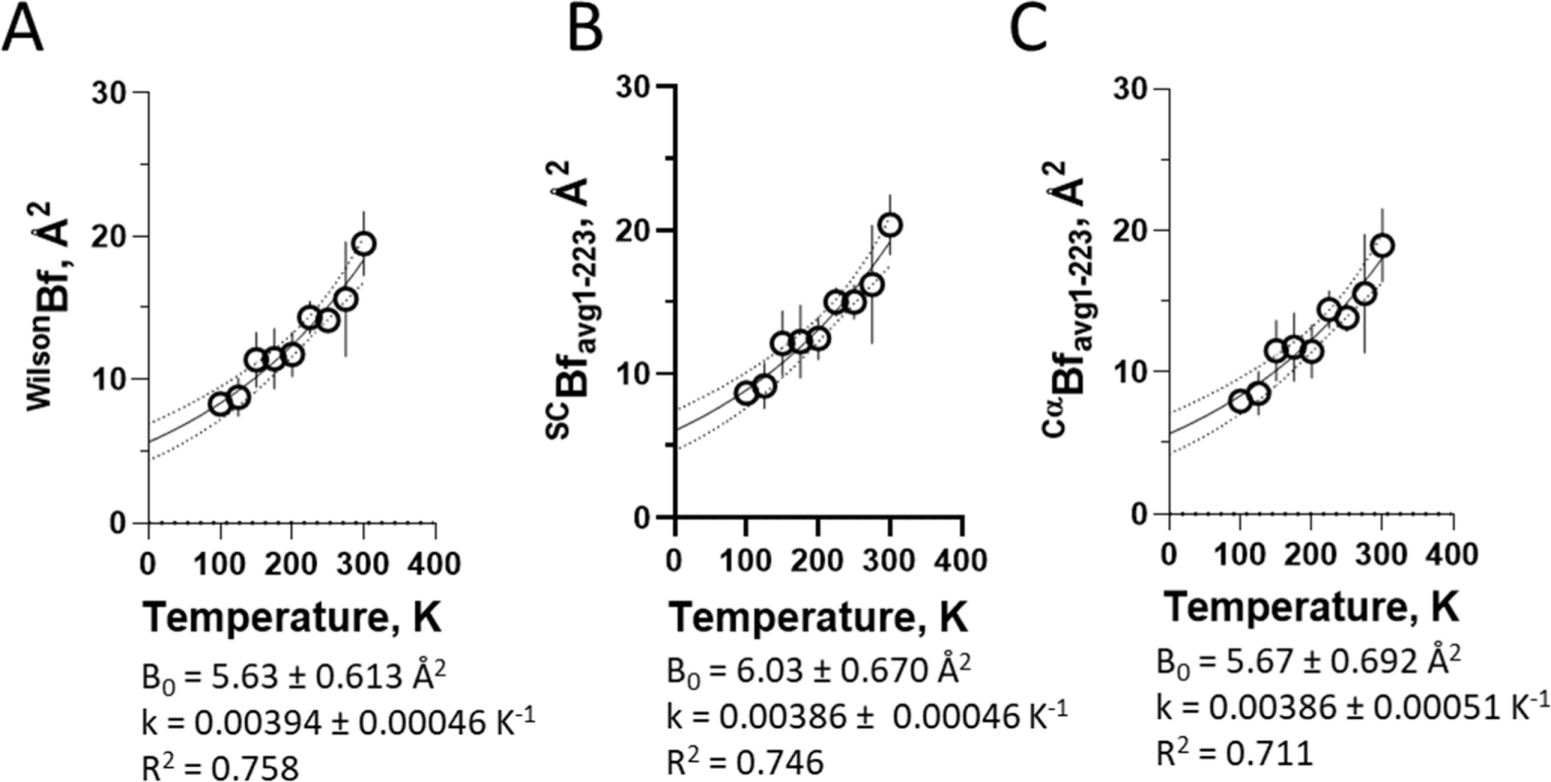
Temperature-dependent changes in B-factor (B). Overall changes in B-factor as a function of temperature for (A) Wilson plot (Pearson r correlation coefficient = 0.9597, with 95% CI 0.8151 to 0.9917), *p*-value two-tailed <0.0001). (B) Side chains (Pearson *r* correlation coefficient = 0.9599, with 95% CI 0.8160 to 0.9918), *p*-value two-tailed <0.0001), and C) Cα (Pearson *r* correlation coefficient = 0.9577, with 95% CI 0.8065 to 0.9913), *p*-value two-tailed <0.0001). Values correspond to the extrapolated B-factor at zero Kelvin (*B*_0_) and the thermal coefficient *k*, as inferred from single exponential (*B*_obs_ = *B*_0_×e^(*k*·*T*)^) nonlinear regression of their respective panels (solid lines; dotted lines are 95% confidence interval). The symbol is the average, and the bar is the standard deviation (*n* = 3).

### Atomic B-Factor Changes by Temperature

The B-factor is variable through the atoms of the system. The B-factor varies broadly along the polypeptide sequence, as seen for side chains (Figure 5A) and Cα (Figure 6A). The B-factor distribution pattern through the polypeptide chain is similar for all temperatures, although at different levels. The variation of the B-factor for a given atom (e.g., Cα, Figure 5B) or a group of atoms (e.g., side chains, Figure 6B) as a function of temperature shows an exponential rise pattern, and the exponential constant shows only minor variability for Cα (Figure 6C) and side chain (Figure 5C) of about 0.005 K^–1^, suggesting a model-free independent behavior of changes in an atomic displacement parameter as a function of temperature.

**Figure 5:**
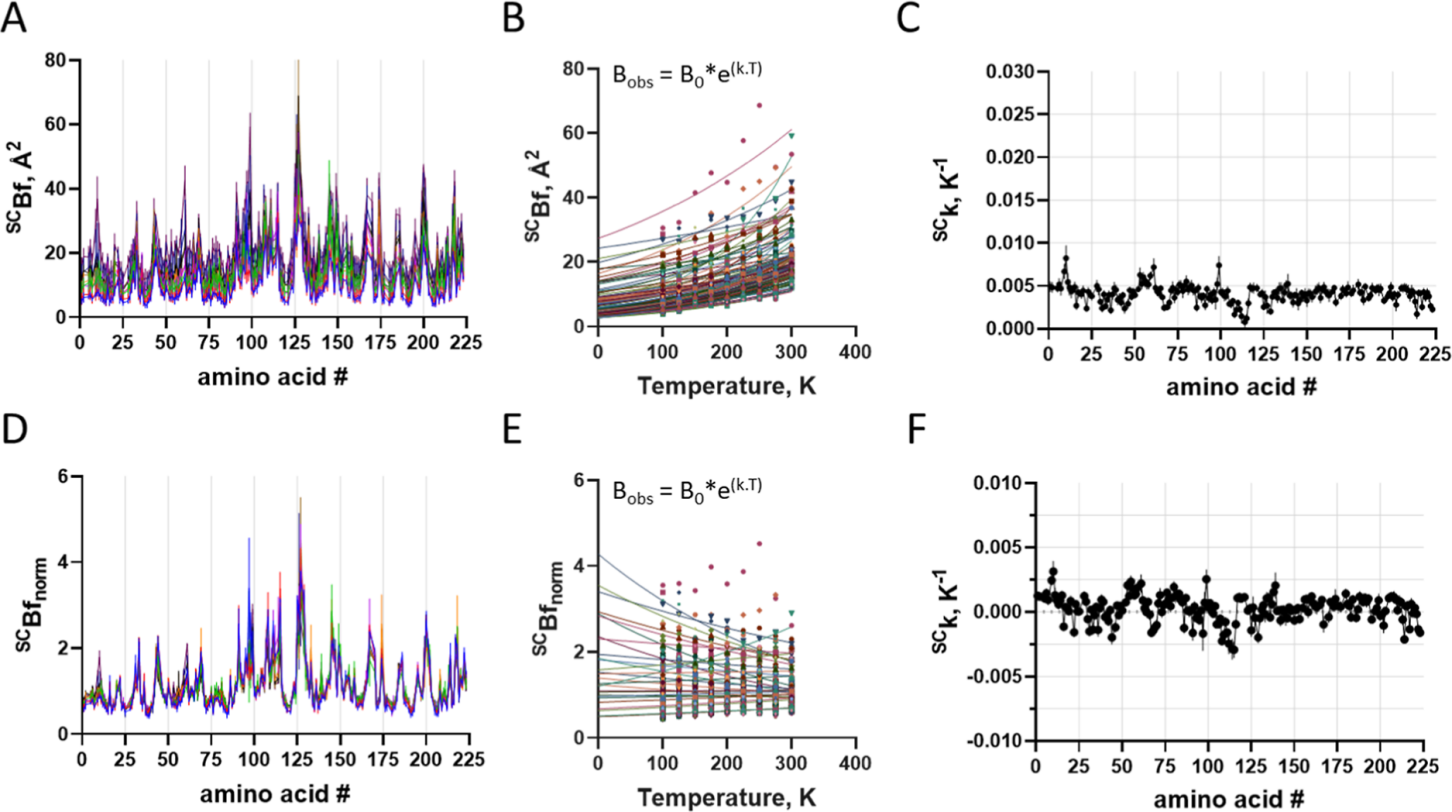
Temperature-dependent changes in the side chain B-factor. (A) B-factor distribution for the side chain at varying temperatures (different colors). (B) Changes in the raw B-factor as a function of temperature for each amino acid side chain (different colors). Lines are exponential nonlinear regression, from which were obtained the raw *B*_0_ and the thermal constant *k*. (C) Distribution of raw *k* along the protein sequence. Pearson *r* correlation coefficient −0.1413 95% CI: −0.2753 to −0.001847), *P* (two-tailed) = 0.0471. (D) Normalized B-factor (*B*_norm_) distribution for the side chain at varying temperatures (different colors). (E) Changes in *B*_norm_ as a function of temperature for each amino acid side chain (different colors). Lines are exponential nonlinear regression, from which was obtained the thermal constant ^norm^ *k*. (F) Distribution of the ^norm^ *k* along protein sequence. Pearson *r* correlation coefficient −0.09924 (95% CI: −0.2354 to 0.04076), *P* (two-tailed) = 0.1642; the symbol represents the average, and the bar represents the standard deviation (*n* = 3).

**Figure 6:**
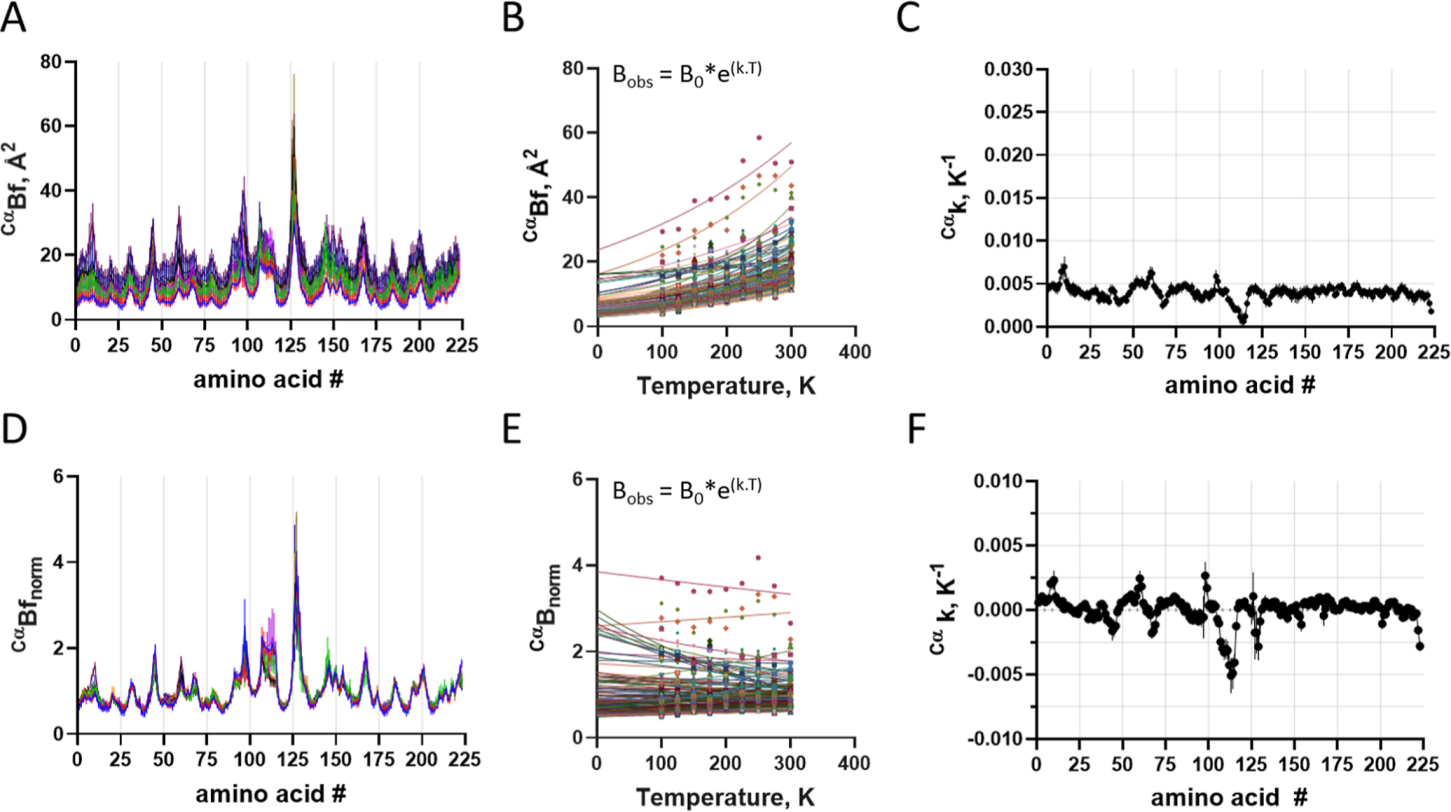
Temperature-dependent changes in the Cα B-factor. (A) B-factor distribution for Cα at varying temperatures (different colors). (B) Changes in raw B-factor as a function of temperature for each amino acid Cα (different colors). Lines are exponential nonlinear regression, from which were obtained the raw *B*_0_ and the thermal constant *k*. (C) Distribution of raw *k* along the protein sequence. Pearson r correlation coefficient −0.1819 (95% CI: −0.3059 to −0.05173), *P* (two-tailed) = 0.0065. (D) Normalized B-factor (*B*_norm_) distribution for Cα at varying temperatures (different colors). (E) Changes in *B*_norm_ as a function of temperature for each amino acid Cα (different colors). Lines are exponential nonlinear regression, from which was obtained the thermal constant ^norm^ *k*. (F) Distribution of ^norm^ *k* along the protein sequence. Pearson *r* correlation coefficient −0.09912 (95% CI: −0.2275 to 0.03268), *P* (two-tailed) = 0.1401. The symbol represents the average, and the bar represents the standard deviation (*n* = 3).

The normalization of the polypeptide B-factor allows a better understanding of local fluctuation. The normalized B-factors for Cα atoms (Figure 6D) and side chains (Figure 5D) resulted in a similar distribution pattern at all temperatures for crystal structure elucidation between 100 K and 300 K. Adjusting the temperature dependence of the normalized B-factor with a single exponential nonlinear regression function according to [eq 5] for Cα (Figure 6E), and side chain (Figure 5E) shows only minor variation, with constant ^norm^ *K* fluctuating around zero for both Cα (Figure 6F) and side chain (Figure 5F), suggesting a lack of local variation of the B-factor as a protein structure feature. A minor variation may be depicted in the amino acid region 108–112, although with no statistical significance (Figure S5) which may be attributed to crystallographic contact (Figure S6).

### Correlation between Conformational Change and B-Factor

A cross-correlation analysis between the B-factor and conformational changes was performed. The variation of the extrapolated zero-point B-factor (zero Kelvin, *B*_0_) for atoms from side chains and Cα were analyzed as a function of temperature-dependent conformational change (Figure 7).

**Figure 7:**
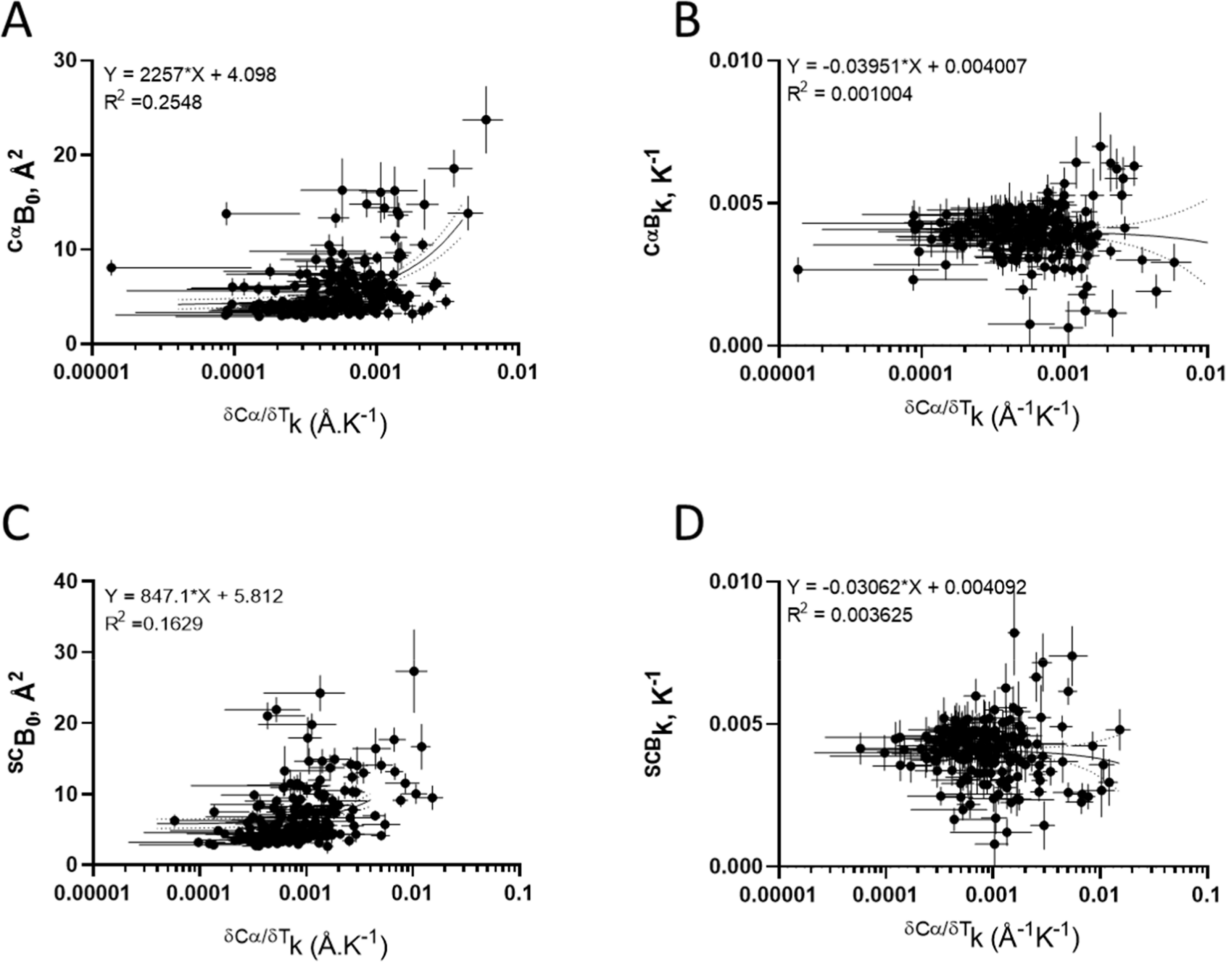
Correlation between B-factor and conformational changes. Correlation between temperature-induced conformational changes constant (δ*SC*/δ*Tk*) and the extrapolated zero-point B-factor (*B*_0_, A,C) or the thermal B-factor dependence constant (*Bk*, B,D) for Cα (A,B) and side chains (SC; C,D). Lines correspond to first-order linear regression (continuous) and 95% confidence interval (dotted). Pearson r correlation coefficient (log *x* scale): (A) *r* = 0.3576 (95% CI: 0.2374 to 0.4670), *P* (two-tailed) < 0.0001; (B) *r* = 0.0228 (95% CI: −0.1089 to 0.1538), *P* (two-tailed) = 0.7344; (C) *r* = 0.4553 (95% CI: 0.3354 to 0.5608), *P* (two-tailed) < 0.0001; and (D) *r* = −0.0761 (95% CI: −0.2154 to 0.06622), *P* (two-tailed) = 0.2941.

The extrapolated zero-point B-factor (*B*_0_, Figure 7A,C) and the thermal B-factor dependence constant (^B^ *k*, Figure 7B,D) is distributed along a broad range of temperature-induced conformational change constant (^δSC/δT^ *k*) and lacking strong correlation between them (Pearson *r* < |0.5|), indicating that the minor local variation found in the B-factor does not associate with the conformational changes induced by temperature.

## Influence of Temperature on the B-Factor of Non-protein Atoms

The effects of temperature on nonprotein atoms were also analyzed. The crystal structure solved at 1.5 Å allowed the determination of a large set of water molecules with a broad distribution of B-factors, ranging from 5 Å^2^ to 60 Å^2^ (Figure 8A). The number of crystallographic water molecules decrease as a function of temperature (Figure 8B), while their respective B-factor increases (Figure 8C), similar to other nonprotein atoms such as calcium (Figure 8E) and benzamidine (Figure 8F,G). The variation in the nonprotein B-factor as a function of temperature was adjusted using a single exponential nonlinear regression function [eq 5], revealing a constant fluctuating at about 0.005 K^–1^ which is in the same magnitude range for all other protein atoms from side chain and Cα. These data reveal a systematic model-free pattern in B-factor change for all atoms of the system as a function of temperature regardless of the nature of the atom.

**Figure 8:**
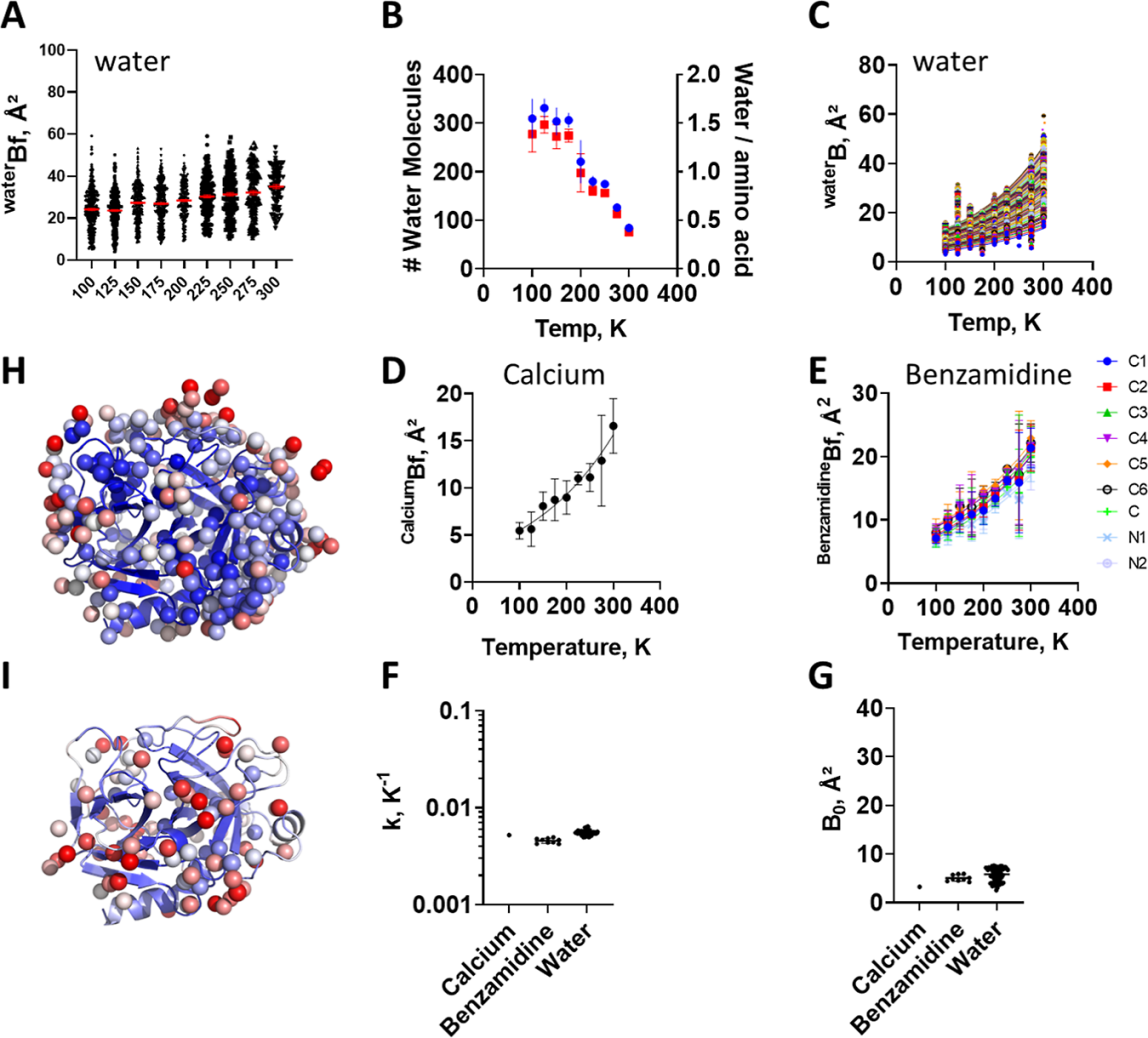
B-factor of nonprotein atoms as a function of temperature. (A) B-factor of the oxygen atoms of the water present in the structure as a function of temperature. (B) Number of water molecules per structure and the water/amino acid quotient. (C) B-factor of all oxygen atoms in conserved water as a function of temperature. Crystallographic structure represented by cartoon colored according to the B-factor (blue-white-red, min 5 Å^2^, max 40 Å^2^), showing water molecules as spheres. Thermal dependence of the B-factor for (D) calcium ions and (E) benzamidine atoms. Lines are exponential nonlinear regression, from which were obtained the thermal constant *k* (F) and the raw *B*_0_ (G) (*k* = δ*B*/δ*T*). (H) PDB: 9AVX and (I) PDB: 9AWU showing the loss of molecules at 300 K. Bars are average and standard error (*n* = 3).

### Analysis of Independent Structures from RCSB

The general effect of temperature and B-factor reported here was searched from independent research groups, in other trypsin–benzamidine structures (Figure S7, Figure S8; Supporting Information), varying in resolution (from 0.75 to 2.4 Å), pH, crystallization conditions, data acquisition, and processing workflows among other differences which bring adequate high methodologic variance for this analysis.

A total of 39 independent crystal structures of bovine trypsin–benzamidine in *P*2_1_2_1_2_1_ were found in the RCSB. These structures were collected either at the cryogenic or room temperature range, revealing a gap between these two temperature ranges (Figure S7A). This gap may be due to individual interest in each of these temperature ranges or due to difficulties in using temperature as a continuous variable in this range. These crystal lattices show close cell parameters, although four of them fall outside the range (Figure S7B,C). The changes in cell parameters showed a positive correlation between **b** vs **a** (Figure S7E) and **c** vs **a** (Figure S7F). These structures show close correlation between data (from Wilson plot of integrated diffraction intensities) and model (final refined structure) B-factors (Figure S7H). These structures show good correlation between *R*_*w*_ and *R*_*f*_, which is minor correlation with the Wilson B-factor.

The B-factor analysis of these structures reported from independent groups showed large variation in the B-factor distribution along the polypeptide chain and conformational change (Figure 9), for both Cα (Figure 9A,C) and side chains (Figure 9D,F). However, upon B-factor normalization, their distribution pattern along the polypeptide chain is in close similarity (Figure 9B,E), indicating that the variations in conformation are not followed by changes in the B-factor. Instead, a dissociation between these two parameters is found.

**Figure 9:**
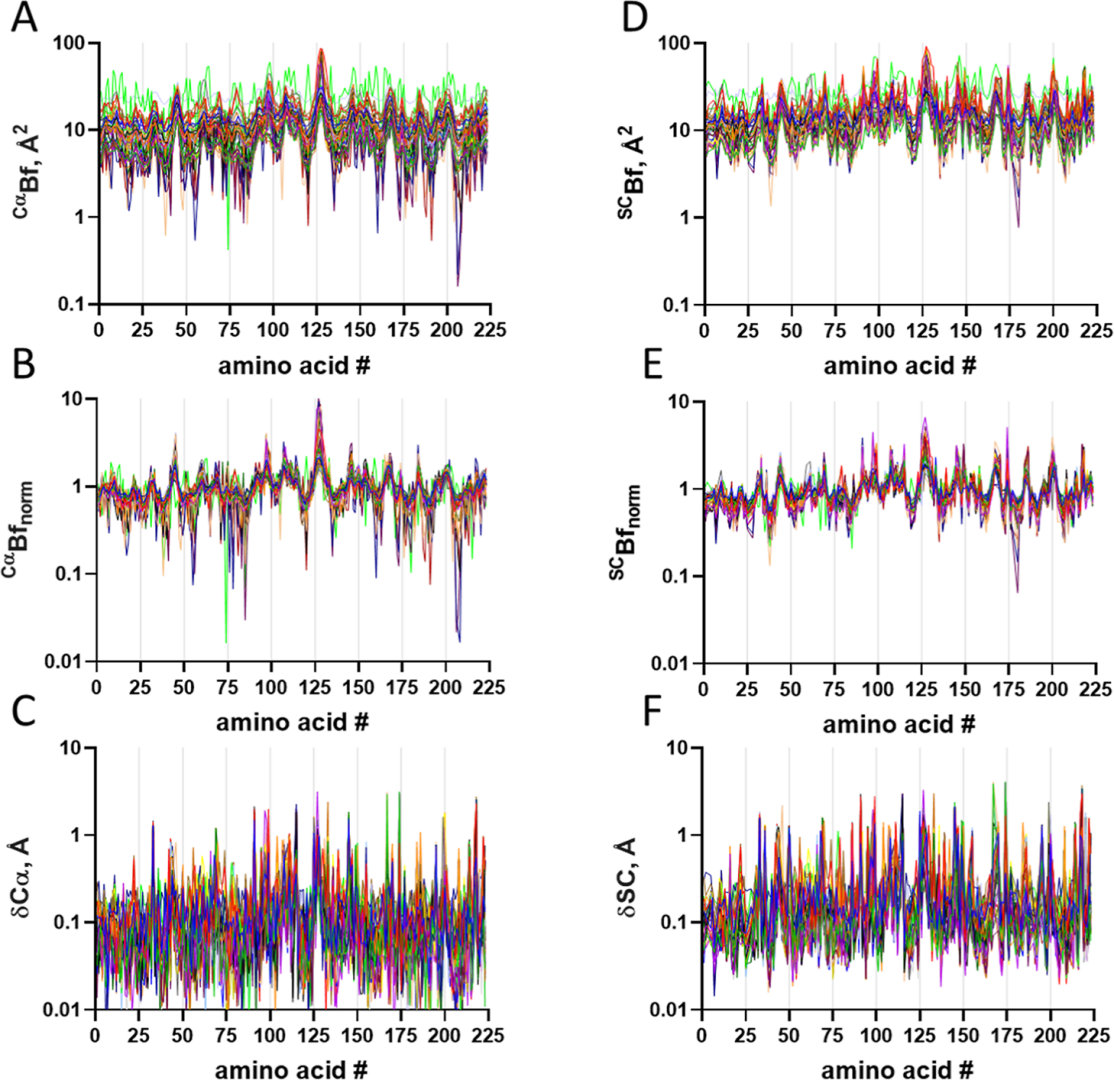
Orthorhombic trypsin with benzamidine in the RCSB Data from the RCSB (access May, 2024) for trypsin in *P*2_1_2_1_2_1_. (A) Distribution of the Cα Raw B-factor for each PDB structure. (B) Distribution of the Cα Norm B-factor for each PDB structure. (C) Distribution of the Cα rmsd for each PDB structure. (D) Distribution of the Side Chain Raw B-factor for each PDB structure. (E) Distribution of the Side Chain Norm B-factor for each PDB structure. (F) Distribution of the side chain rmsd for each PDB structure.

Together, our present data provide evidence that crystal stabilization allows reproducible measurements in a broad temperature range from cryo to room temperature. Moreover, from present data and independent data from other research groups, we have found that while physical and chemical variables (including temperature and pH crystallization conditions) can result in modifications in the crystallographic B-factor, this parameter does not seem to correlate with conformational changes.

## Discussion

In this study, we report a structural analysis of trypsin in complex with benzamidine solved at varying temperatures over a 200 K interval, providing insights into protein conformational diversity and its relationship with the crystallographic B-factor. Local conformational changes induced by temperature follow a nonlinear pattern, which does not correlate with their respective atomic B-factors and temperature-dependent changes. While the B-factors provide insight into the mean-square displacement of atoms, they do not directly correlate with local conformational changes. Such dissociation between conformation and B-factors is also found in other structural studies, emphasizing the complex nature of conformational flexibility and also the contributors to the average B-factor.

The three “Rs” that comprise high-quality scientific research are known as rigor, reproducibility, and robustness.^22^ Achieving reproducibility in B-factor measurements has been proven to be challenging, hindering the understanding of each factor’s specific contribution. To address this issue, we first proposed a mathematical normalization,^19^ which revealed the high reproducibility of B-factors between crystals collected at the same temperature by different instruments, analysts, and data processing workflows and can assist ensemble refinement.^23–25^

Our normalization method, already incorporated into practice,^23,26^ did not solve the reproducibility issue regarding the raw B-factor and did not explain the differences between similar crystals in the same or different instruments. Recently, we demonstrated the use of hydrocarbon grease to protect lysozyme protein crystals for data collection over a broad temperature range (cryogenic to 325 K) at high resolution (1.5 Å) in triplicate.^20^ Protective measures against dehydration for single-temperature data collection have been shown using specific devices^21^ or embedding the crystals into hydrophobic chemicals, such as lipid cubic phase (LCP),^27^ oils,^28–31^ mineral oil,^32^ shortening,^33^ and fat.^34,35^ This technique shows potential for reproducible measurements using temperature as a physical variable, which allowed us to find a lack of correlation between B-factor changes and temperature-induced conformational transitions using both normalized and raw B-factors.^20^

Temperature effects on nonprotein atoms were also investigated. Initially, a broad distribution of B-factors for water molecules was observed. While the number of crystallographic water molecules decreased with temperature, the B-factors of such remaining water molecules increased uniformly as a function of temperature, as also observed for protein atoms, indicating consistent behavior across different atom types. Similarly, other nonprotein atoms such as calcium and the benzamidine molecule exhibited similar effects on the B-factor as a function of temperature, providing evidence that the temperature-induced changes in B-factors are uniform for all atoms of the system. The thermal expansivity constants for nonprotein atoms were comparable, reinforcing the homogeneous impact of temperature across various atomic components analyzed in the study. Analysis conducted with two refinement programs resulted in similar thermal dependencies for the B-factors (Refmac—Figures 4, 6, and 7; and Phenix.refine—Figure S9, S10, and S11). These findings provide a cross-validation of the thermal response of B-factors, corroborating it as an intrinsic physical propensity of the crystalline system, reflecting global rigid-body vibrations rather than software-specific refinement biases.

The analysis of RCSB entries for trypsin–benzamidine crystal structures from other groups revealed heterogeneous conformations, while similar in the normalized B-factors. This result has multiple interpretations: first, a demonstration of the importance of elucidating crystal structures under varying chemical and physical conditions in order to explore the conformational space that can be populated and second the robustness of B-factor normalization for comparative structural analysis since each data originates from dissimilar workflows and softwares (integration: iMosFlm, XDS, Xia2, DENZO, PROTEUM3, HKL-2000, and CrysAlisPro; and refinement: Refmac, Phenix, SHELX, and CNS). Finally, there is a lack of correlation between changes in conformation and B-factor.

In our crystallographic examination of the temperature effect on the trypsin–benzamidine enzyme–ligand complex, we found no discernible correlation between temperature-induced conformational changes and B-factors. The B-factor variation due to the temperature remained consistent across all atoms within the system. These findings suggest a detachment between the absolute B-factor values and conformational plasticity within this particular system. The similar thermal dependence of B-factors for all atoms suggests a major contribution from uniform rigid-body vibration of the whole system rather than localized flexibility.

The pioneering work of Fraudenfelder, Petsko, and Tsernoglou with crystal structures of myoglobin solved from data collected at temperatures ranging from 220 to 300 K demonstrated the thermal dependence of the B-factor on temperature, although not allowing separation between the vibrational (*X* ^2^_v_) and rigid-body (*X* ^2^_RB_) terms.^15,16^ In our present work, we could determine that the rigid-body vibration contributes with a single exponential constant of 0.005 K^–1^ (eq 5), while the zero-point B-factor (*B*_0_) may show contributions from conformational substates, lattice contacts and disorder, and atomic and local (conformational) vibration. In this context, the lack of correlation between conformational changes and zero-point B-factor suggests that either the absolute or rescaled B-factor does not seem to be the best approach for inferring dynamics and conformational plasticity.

The interpretation of the crystallographic B-factor as an indicator of local protein flexibility is tempting and remains pervasive in structural biology literature.^36^ However, this conceptual link—between B-factor amplitude and conformational mobility—has not been supported by experimental validation. Instead, the dissociation between these two variables has originally been demonstrated by Prof. Christopher Dobson and colleagues, using lysozyme and dynamic measurements by NMR.^17^ Using primary crystallographic data of lysozyme and insulin from our group and secondary, independent data from other groups, we have additionally shown that variations in the B-factor do not correlate with local conformational transitions.^19,20^ In the present work, using trypsin–benzamidine as an independent structural model, we confirmed and extended these findings on the dissociation of B-factor and conformational plasticity, while adding evidence for the uniform, all-atom variation in the B-factor as a function of temperature, most likely due to whole-system rigid body motion. In conjunction, all of these independent data from distinct proteins indicate that B-factors, either the raw values or rescaled, may not be the best surrogates for protein dynamics. The generality of these observations across structurally unrelated proteins and under distinct crystallographic conditions argues for a re-evaluation of the common assumptions linking B-factors to functional flexibility. Drawing the folding landscape of proteins, their dynamics and conformational plasticity may be better accessed by techniques such as multiple conformers from serial crystallography, multiple single-crystal structures from independent data sets, use of chemical (e.g., pH, salts) and/or physical (temperature, pressure) variables, and molecular dynamics by simulation, normal modes, and/or NMR.^37,38^ Solving multiple structures from independent data sets and using varying techniques may provide access to the understanding of conformational plasticity and polymorphism and the understanding of the folding landscape of proteins.

## Conclusions

Our data establish that hydrophobic embedding stabilizes crystals for reproducible B-factor analysis across temperature ranges from cryogenic to room and higher temperatures while revealing that B-factors predominantly reflect global vibrations rather than local dynamics. This necessitates re-evaluating their use as flexibility proxies. We advocate for integrative approaches combining ensemble crystallography, MD simulations, and thermodynamic analyses to decipher conformational landscapes.

## Supplementary Material





## Data Availability

The experimental information and data supporting the findings of this study are available within the paper and the indicated data repository, under PDB ID listed in Table S1 (9AVX, 9AVY, 9AVZ, 9AW0, 9AW1, 9AW2, 9AW4, 9AW8, 9AW9, 9AWA, 9AWB, 9AWC, 9AWD, 9AWF, 9AWG, 9AWH, 9AWI, 9AWL, 9AWM, 9AWN, 9AWO, 9AWP, 9AWQ, 9AWR, 9AWS, 9AWU, 9AWV, and 9AWZ). Further information is available from the corresponding authors upon reasonable request.

## Notes

Thermal-dependent changes in unit cell parameters; thermal-dependent conformational changes in trypsin; Pearson correlation between changes in trypsin conformation and temperature; Pearson correlation between changes in trypsin B-factor and temperature; representation of the crystal contact generated by the symmetry found in the P212121 space group; orthorhombic trypsin–benzamidine structures in the RCSB; alignment of trypsin–benzamidine complexes; temperature-dependent changes in B-factor (B); temperature-dependent changes in Cα B-factor; correlation between B-factor and conformational changes; and structural and statistical analysis (PDF)
Crystallographic information on raw data collection and refinement statistics from this work and orthorhombic (*P*2_1_2_1_2_1_) data of trypsin crystals with benzamidine, determined via single-crystal X-ray diffraction, retrieved from The Research Collaboratory for Structural Bioinformatics Protein Data Bank (RCSB PDB, rcsb.org) as of May 2024 (XLSX)

## Abbreviations

CD: circular dichroism.
