# Supplementary material for: Temperature-Resolved Crystallography Reveals Rigid-Body Dominance over Local Flexibility in B‑Factors
Source: ACS Omega. 2025 Aug 20;10(34):38871–81. doi: 10.1021/acsomega.5c04454 (PMC12409559; doi:10.1021/acsomega.5c04454)
Supplement: Supplementary file 1 [file ao5c04454_si_001.pdf]

Supporting Information

Temperature-Resolved Crystallography Reveals Rigid-Body Dominance Over Local Flexibility in B-Factors

Fernando de Sá Ribeiro[1,2] and Luís Maurício T. R. Lima[1,2,3,4*]

[1] Laboratório de Biotecnologia Farmacêutica (pbiotech), Faculdade de Farmácia, Universidade Federal do Rio de Janeiro, Rio de Janeiro, RJ, 21941-902, Brazil.
[2] Programa de Pós-Graduação em Química Biológica, Universidade Federal do Rio de Janeiro, Rio de Janeiro, RJ, 21941-902, Brazil.
[3] Programa de Pós-Graduação em Ciências Farmacêuticas, Faculdade de Farmácia, Universidade Federal do Rio de Janeiro, Rio de Janeiro, RJ, 21941-902, Brazil.
[4] Programa de Pós-Graduação em Nutrição, Universidade Federal do Rio de Janeiro, Rio de Janeiro, RJ, 21941-902, Brazil.

Running title: Thermal-dependence of B-factor

*To whom correspondence should be addressed

Authors Address and Contact
- Luis Mauricio T. R. Lima – Laboratório de Biotecnologia Farmacêutica (pbiotech), Faculdade de Farmácia, Universidade Federal do Rio de Janeiro – UFRJ, CCS, Bss24, Ilha do Fundão, 21941-590, Rio de Janeiro, RJ, Brazil. Phone/Fax: (+55-21) 3938-6639 – LuisMauricioLima@gmail.com, Mauricio@pharma.ufrj.br. Social Media: @pbiotech

AUTHOR LIST
Fernando de Sá Ribeiro – FernandoRibeiroBiomed@gmail.com
Luís Maurício T. R. Lima – LuisMauricioLima@gmail.com , Mauricio@pharma.ufrj.br

Contents

Figure S1. Thermal-dependent changes in unit cell parameters. 3

Figure S2. Thermal-dependent changes in unit cell parameters. 4

Figure S3. Thermal-dependent conformational changes in trypsin. 5

Figure S4. Pearson correlation between changes in trypsin conformation and temperature 6

Figure S5. Pearson correlation between changes in trypsin B-factor and temperature 7

Figure S6. Representation of the crystal contact generated by the symmetry found in the $P2_12_12_1$ space group. 8

Figure S7. Orthorhombic trypsin-benzamidine structures in the RCSB 9

Figure S8. Alignment of trypsin-benzamidine complexes from the RCSB. Data from the RCSB (access May, 2024) for trypsin-benzamidine in $P2_12_12_1$. 10

Figure S9. Temperature-dependent changes in B-factor (B). 11

Figure S10. Temperature-dependent changes in Cα B-factor. 12

Figure S11. Correlation between B-factor and conformational changes. 13

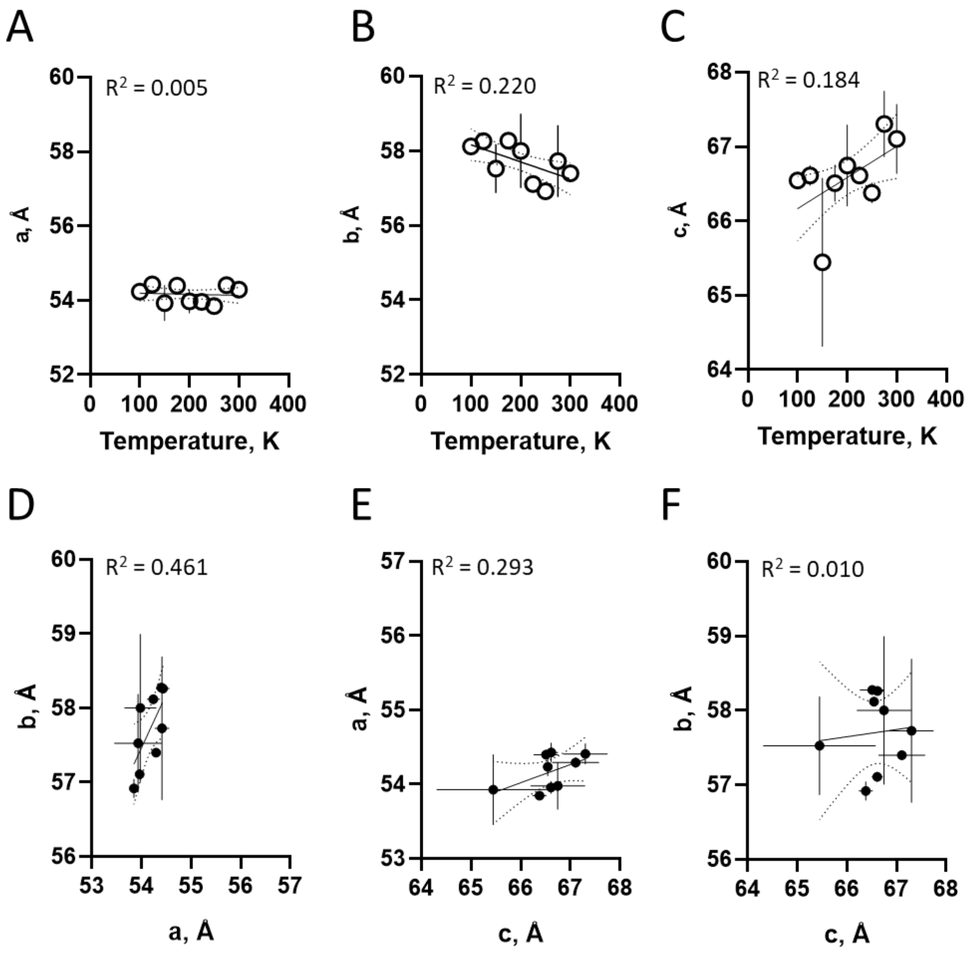

Figure S1. Thermal-dependent changes in unit cell parameters.

Correlation between cell unit parameters and data collection temperature for single crystal x-ray diffraction of Trypsin in $P2_12_12_1$. Continuous lines are first order linear regression and dotted lines are 95 % confidence interval. The linear correlation between data collection temperature

A) a as a function of temperature,
B) b as a function of temperature,
C) c as a function of temperature,
D) b as a function of a,
E) a as a function of c and
F) b as a function of c.
Symbol is average and bar is standard deviation (n=3).

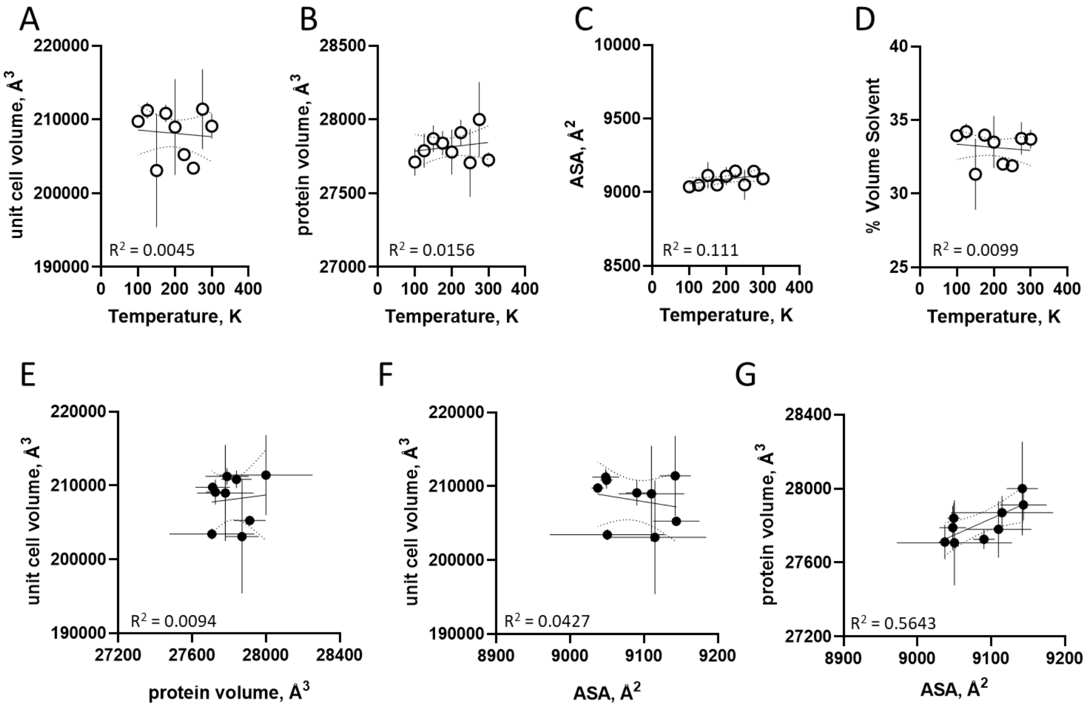

Figure S2. Thermal-dependent changes in unit cell parameters.

Correlation between cell unit parameters and data collection temperature for single crystal x-ray diffraction of Trypsin in $P2_12_12_1$. Continuous lines are first order linear regression and dotted lines are 95 % confidence interval. The linear correlation between data collection temperature
A) Unit cell volume a function of temperature,
B) protein volume as a function of temperature,
C) Accessible surface area (ASA) as a function of temperature,
D) % volume solvent as a function of a,
E) Unit cell volume a as a function of protein volume,
F) Unit cell volume a as a function of ASA and
G) Protein volume a as function of ASA.
Symbol is average and bar is standard deviation (n=3).

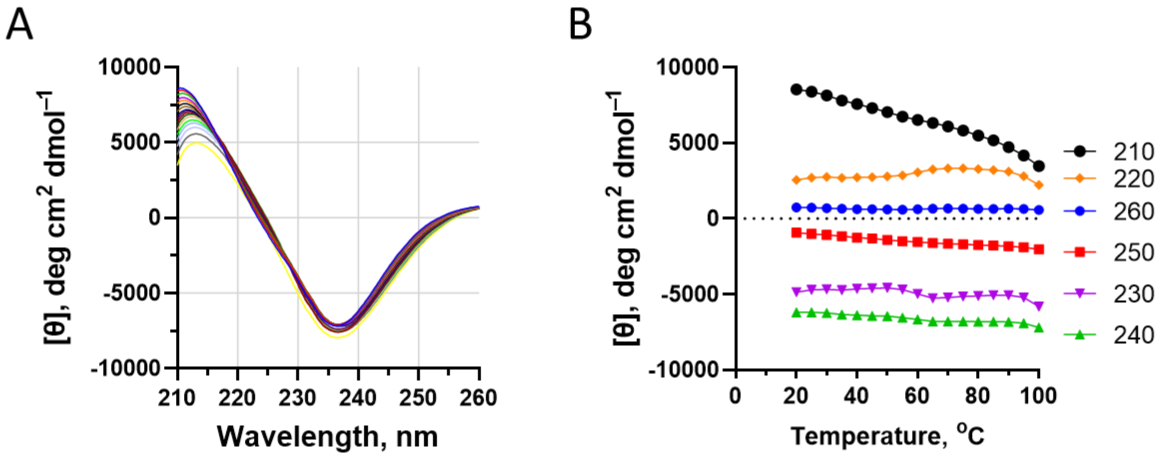

Figure S3. Thermal-dependent conformational changes in trypsin.

Temperature effect on lysozyme was monitored by circular dichroism providing information regarding secondary structure.

A) Circular dichroism spectra of trypsin at temperatures from 20 °C to 100 °C in 5 °C intervals;

B) Thermal curves at varying wavelengths (data from Fig. S3A; average and standard deviation from 0.4 degree intervals);

Measurements were performed with trypsin at 5 mg/mL in 20 mM dibasic potassium phosphate with benzamidine.

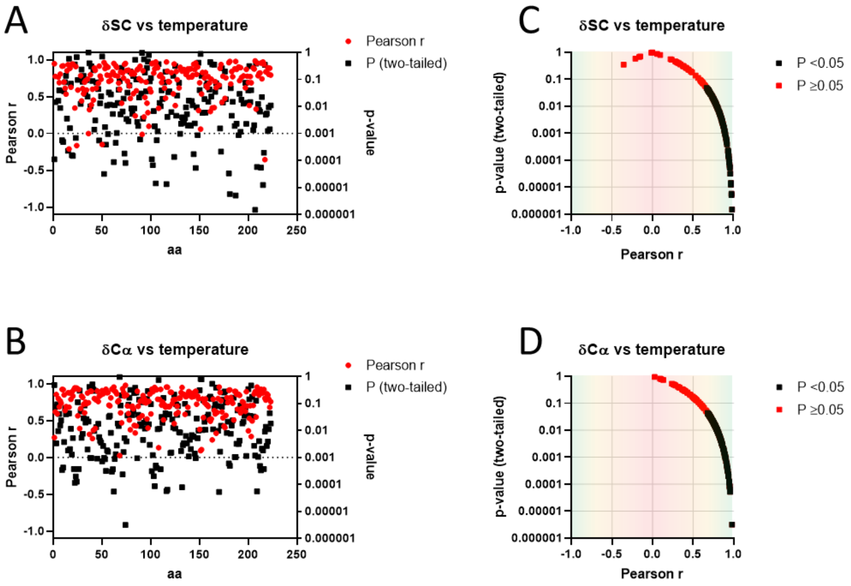

Figure S4. Pearson correlation between changes in trypsin conformation and temperature

Changes in side-chain (Fig. 2) and Cα (Fig. 3) conformation were plotted as a function of temperature and analyzed for Pearson correlation.

The Pearson r and p-value (two-tailed) were plotted as a function of trypsin sequence (continuous numbering) for (A) side-chain ($r>0.67$ for $p<0.05$) and (B) Cα ($r>0.68$ for $p<0.05$).

Notice a correlation between Pearson r and p-value for both (C) side-chain and (D) Cα. Most data lie with $r>0.5$ and $p<0.05$, suggesting strong positive correlation.

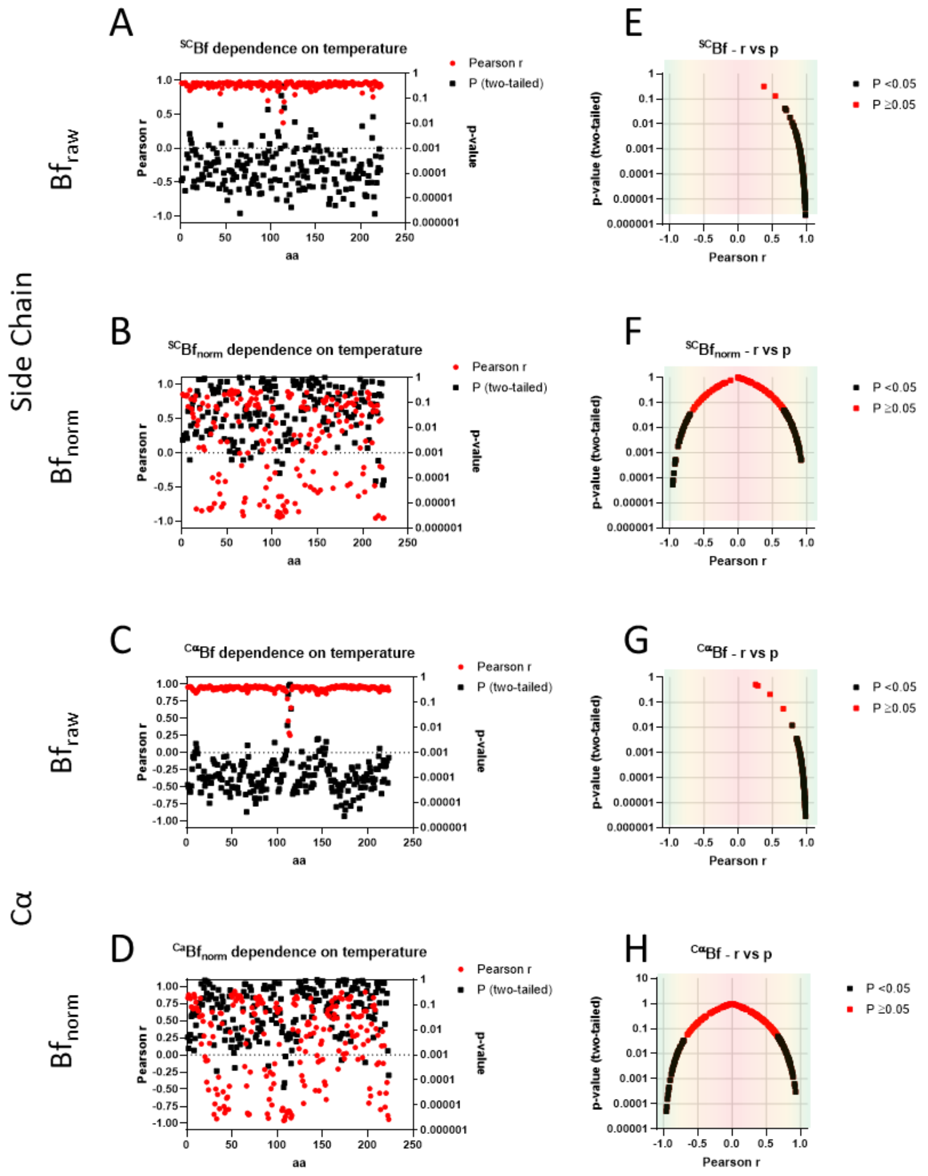

Figure S5. Pearson correlation between changes in trypsin B-factor and temperature

Changes in side-chain (Fig. 5) and Cα (Fig. 6) B-factors were plotted as a function of temperature and analyzed for correlation.

The Pearson r and p-value (two-tailed) were plotted as a function of trypsin sequence (continuous numbering) for side chains (A, B) and Cα (C, D).

Notice a correlation between Pearson r and p-value for both

(E, F) side-chain (Bf_{raw}, $r>0.68$ for $p<0.05$; Bf_{norm}, $r>|0.67|$ for $p<0.05$) and

(G, H) Cα (Bf_{raw}, $r>0.78$ for $p<0.05$; Bf_{norm}, $r>|0.67|$ for $p<0.05$).

Most data for raw B-factor lies with $r>0.8$ and $p<0.05$ (E, G), suggesting strong positive correlation, while normalization (F, H) reduces correlation.

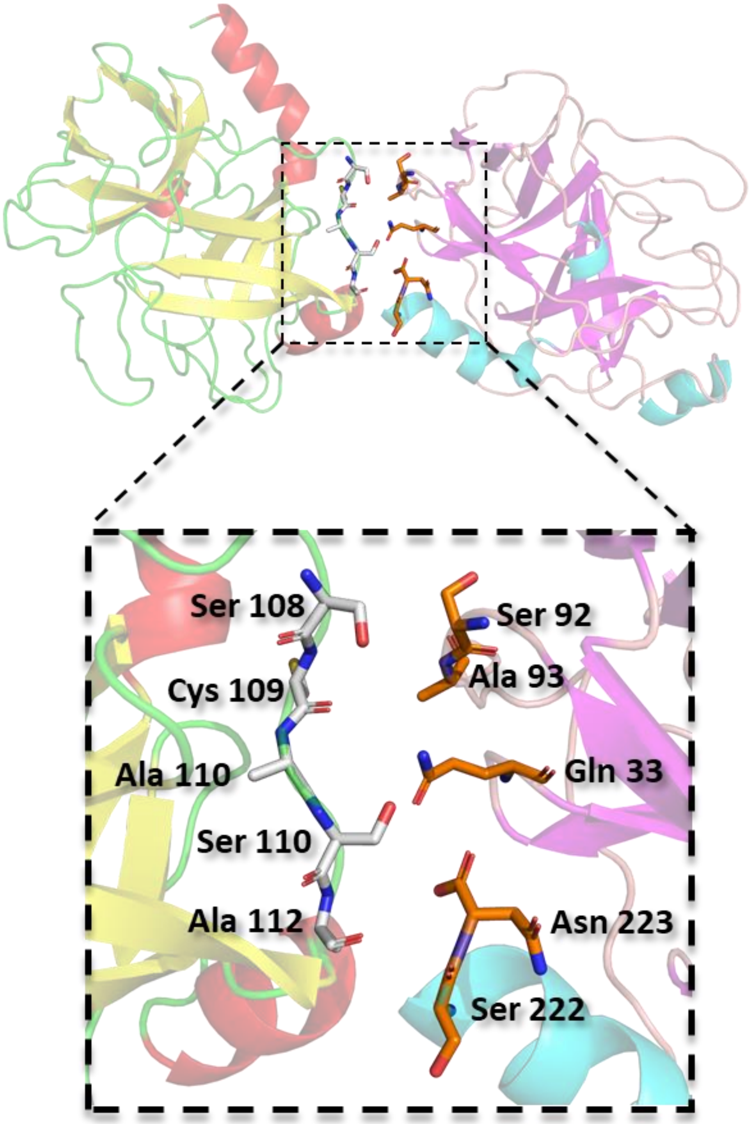

Figure S6. Representation of the crystal contact generated by the symmetry found in the $P2_12_12_1$ space group.
The structure of PDB: 9AVX is represented in cartoon with colors corresponding to secondary structures: yellow for alpha helices, red for beta sheets, and green for coils. The residues important for crystal contact are represented by sticks and colored according to elements: gray for carbon, blue for nitrogen, red for oxygen, and yellow for sulfur.
The symmetry-generated structure produced by PyMOL within a distance of 4 Å is represented in cartoon with colors corresponding to secondary structures: cyan for alpha helices, purple for beta sheets, and pink for coils.
The residues important for crystal contact are represented by sticks and colored according to elements: orange for carbon, blue for nitrogen, red for oxygen, and yellow for sulfur.
The residues important for the contact are labeled with their three-letter codes and respective positions.

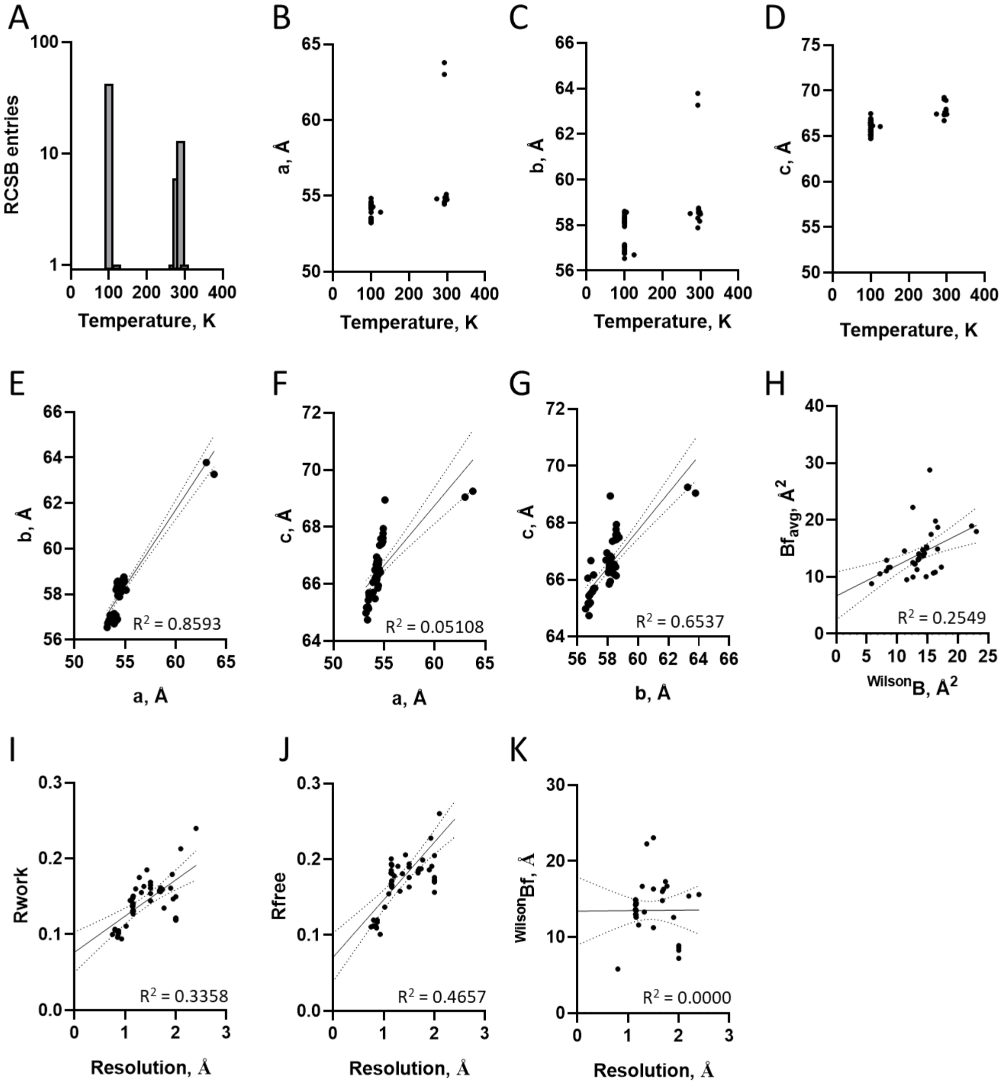

Figure S7. Orthorhombic trypsin-benzamidine structures in the RCSB

Data from the RCSB (access May, 2024) for trypsin-benzamidine in $P2_12_12_1$.

A) Distribution of entries according to data collection temperature, found between 100 k to 300 K.

B) Unit cell length in the a axis as a function of temperature.

C) Unit cell length in the b axis as a function of temperature.

D) Unit cell length in the c axis as a function of temperature.

Correlation between data collection temperature (from 100 k to 300 k); (E) unit cell length in the b axis as a function of a, (F) unit cell length in the b axis as a function of a (G) unit cell length in the c axis as a function of b, H) Correlation between B-factors (all trypsin with benzamidine $P2_12_12_1$) from Wilson estimate and average from final structure model (39 entries). (I) distribution of Rwork and Rfree for deposited structures.

Correlation between data collection temperature (from 100 k to 300 k); (J) Rfree as a function of Rwork, (K) Rfree as a function of Wilson and (L) Rfree as a function of Rwork

Continuous lines are first order linear regression and dotted lines are 95 % confidence interval.

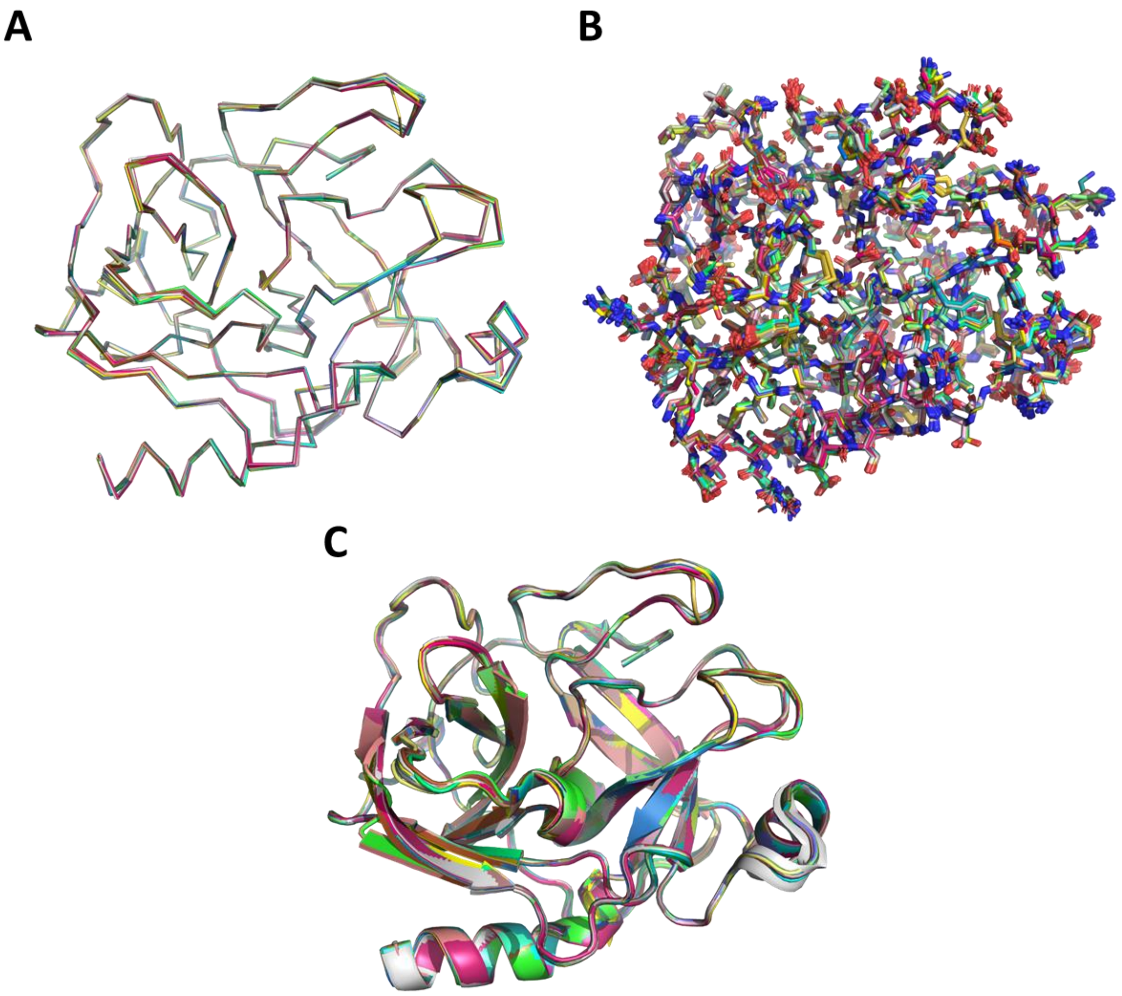

Figure S8. Alignment of trypsin-benzamidine complexes from the RCSB. Data from the RCSB (access May, 2024) for trypsin-benzamidine in $P2_12_12_1$.

A) backbone,

B) side chains and

C) cartoon

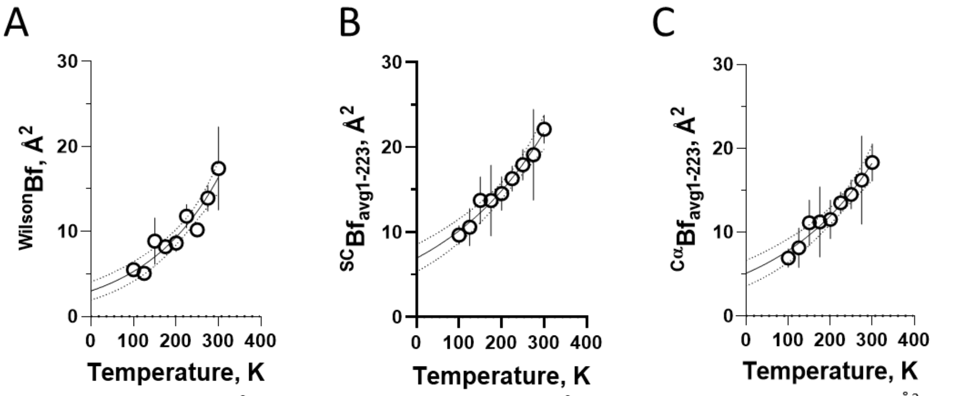

A) B_0 = 3.01 ± 0.52 Å^2
k = 0.00565 ± 0.00069 K^{-1}
R^2 = 0.753

B) B_0 = 6.94 ± 0.77 Å^2
k = 0.00382 ± 0.00047 K^{-1}
R^2 = 0.742

C) B_0 = 5.10 ± 0.736 Å^2
k = 0.00425 ± 0.00060 K^{-1}
R^2 = 0.700

Figure S9. Temperature-dependent changes in B-factor (B).

Overall changes in B-factor as a function of temperature for
A) Wilson plot
B) Side-chains
C) Cα
Values corresponds to the extrapolated B factor at zero Kelvin (B_0) and the thermal coefficient k, as inferred from single exponential ($\mathbf{B_{obs} = B_0 * e^{(k.T)}}$) non-linear regression of their respective panels (solid lines; dotted lines are 95% confidence interval). Symbol is average and bar is standard deviation (n=3). Data were obtained from refinement conducted with Phenix.refine in default mode (https://www.phenix-online.org/).

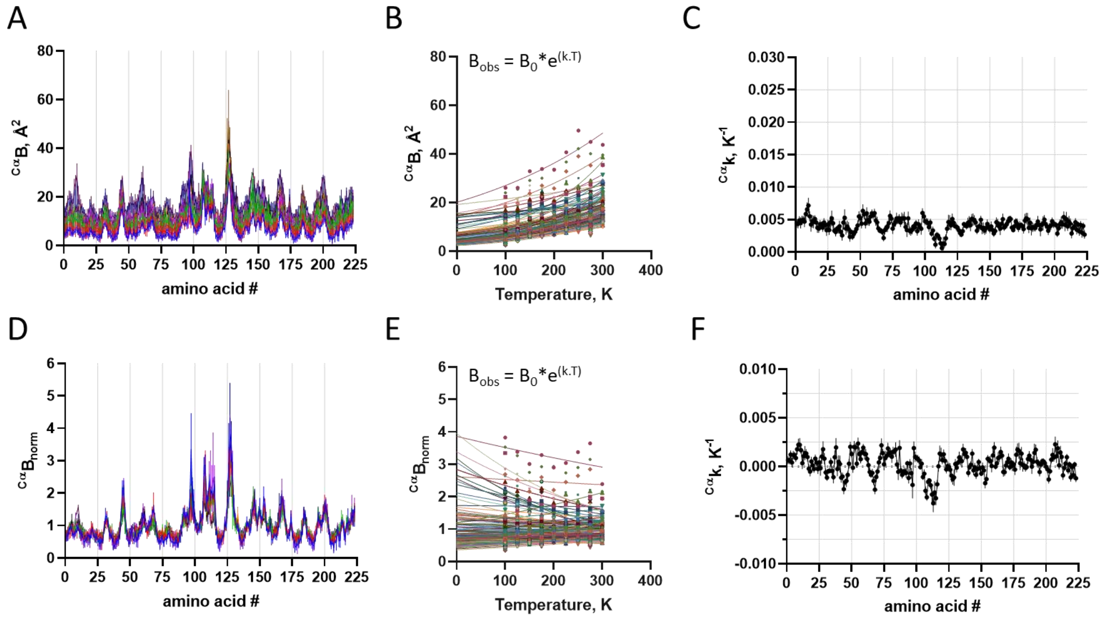

Figure S10. Temperature-dependent changes in Cα B-factor.

A) B-factor distribution for Cα at varying temperatures (different colors).

B) Changes in raw B-factor as a function of temperature for each amino acid Cα (different colors). Lines are exponential non-linear regression, from which were obtained the raw B_0 and the thermal constant k.

C) Distribution of raw k along protein sequence.

D) Normalized B-factor (B_{norm}) distribution for Cα at varying temperatures (different colors).

E) Changes in B_{norm} as a function of temperature for each amino acid Cα (different colors). Lines are exponential non-linear regression, from which was obtained the thermal constant ^{norm}k.

F) Distribution of ^{norm}k along protein sequence.

Symbol is average and bar is standard deviation (n=3). Data were obtained from refinement conducted with Phenix.refine in default mode (https://www.phenix-online.org/).

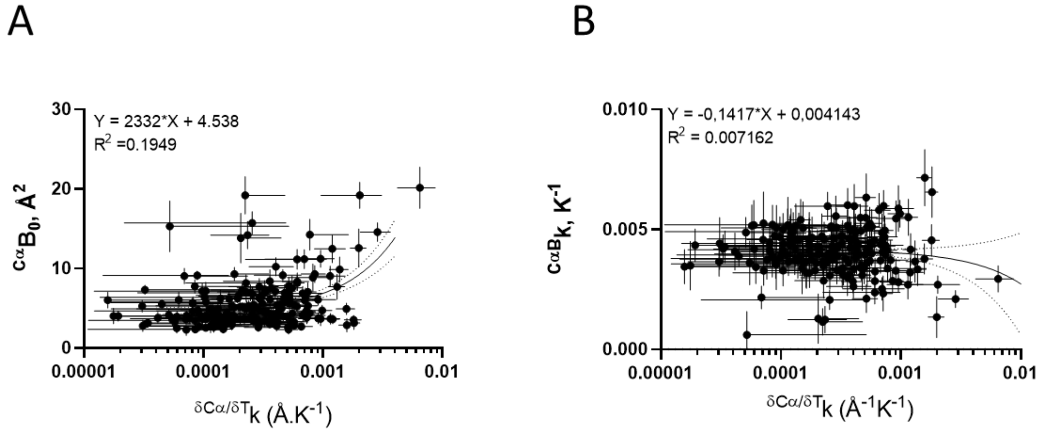

Figure S11. Correlation between B-factor and conformational changes.

Correlation between temperature-induced conformational changes constant ($^{\delta SC/\delta T}k$) and the extrapolated zero-point B-factor (B_0, **A**) or the thermal B-factor dependence constant (^{B}k, **B**) for Cα. Lines correspond to first-order linear regression (continuous) and 95 % confidence interval (dotted). Data were obtained from refinement conducted with Phenix.refine in default mode (https://www.phenix-online.org/).
